# Maximal mouth opening is a simple method to evaluate the treatment outcome of temporomandibular joint arthritis in patients with juvenile idiopathic arthritis

**DOI:** 10.2340/aos.v83.42438

**Published:** 2024-12-18

**Authors:** M. Huhtanen, K. Mikola, A. Kiukkonen, T. Palotie

**Affiliations:** a Orthodontics, Department of Oral and Maxillofacial Diseases, Clinicum, Faculty of Medicine, University of Helsinki, Helsinki, Finland; b Department of Oral and Maxillofacial Diseases, Helsinki University Hospital, Helsinki, Finland; c Oral Health Care, Espoo Health Care Centre, Western Uusimaa Wellbeing Services County, Espoo, Finland; d New Children’s Hospital, Paediatric Research Centre University of Helsinki and Helsinki University Hospital, Helsinki, Finland

**Keywords:** Juvenile idiopathic arthritis, temporomandibular joint arthritis, magnetic resonance imaging, intra-articular corticosteroid injections

## Abstract

**Objective:**

Temporomandibular joint (TMJ) arthritis is a common finding in juvenile idiopathic arthritis (JIA) patients. TMJ arthritis can cause significant disturbances in TMJ function and growth without treatment. Our aim was to evaluate the effectiveness of medical treatments used to manage TMJ arthritis and how to evaluate the outcome of the treatment. Furthermore, this study aimed to ascertain the prevalence of TMJ arthritis in JIA patients and investigate the potential impact of specific factors.

**Material and methods:**

Between 2015 and 2019, a total of 194 JIA patients who received treatment at the Department of Oral and Maxillofacial Diseases, Helsinki University Hospital, Finland were included in the study. We retrospectively screened the patient records and imaging studies to find out how many patients had TMJ arthritis and what medication was used to treat it.

**Results:**

Maximal incisal mouth opening (MIO) increased significantly with patients whose TMJ arthritis was successfully treated with intra-articular corticosteroid injection (IACI). Almost all patients with TMJ arthritis were treated with an IACI at some point during their treatment. Overall, 99 patients (51%) had been diagnosed with TMJ arthritis. No statistical difference was found between the prevalence of TMJ arthritis and different JIA subtypes, JIA onset time, gender, or immunological factors.

**Conclusion:**

MIO is an easy way to evaluate the treatment outcome and possible disease activation of TMJ arthritis. The prevalence of TMJ arthritis is high among JIA patients. In our study, we could not find any parameters that predict TMJ arthritis, and despite systemic medication, TMJ arthritis might occur.

## Background

Juvenile idiopathic arthritis (JIA) is a chronic inflammatory disease characterized by arthritis affecting one or more joints, disease onset before the age of 16 years, and symptoms lasting more than 6 weeks [1, 2]. The incidence of JIA in Finland is reported to be 21/100,000 [3] and it has a female predominance, 3:1 [1, 4]. The latest JIA classification is defined by the International League Association for Rheumatology (ILAR) and includes the following subtypes: oligoarthritis, polyarthritis rheumatoid factor-negative (RF–) and rheumatoid factor-positive (RF+), psoriatic arthritis, enthesitis-related arthritis, undifferentiated arthritis, and systemic arthritis [5].

Temporomandibular joint (TMJ) arthritis is a common manifestation of JIA in all subtypes [6–10], prevalence varying from 34% to 43% [11–13]. TMJ arthritis can include orofacial symptoms: a decreased and deviated mouth opening, pain while eating, and temporomandibular dysfunction (TMD), but can also remain asymptomatic. Maximal incisal mouth opening (MIO) is easily measured by a healthcare professional with a ruler.

A lack of clinical signs might delay diagnosis, leading to condylar resorption and the destruction of the growing mandibular condyle, which interferes with the growth of the lower jaw and leading to severe malocclusions [10, 14–17]. Condylar resorption might occur in all JIA subtypes, but it has been reported more in patients with polyarticular disease, and where disease onset is recorded at a young age [18, 19]. In addition, antinuclear-antibody-positive (ANA-positive) patients seem to be at greater risk of condylar resorption, while human leukocyte antigen B27 (HLA-B27) positivity might be a protective factor [18].

The primary goal in treating JIA is achieving drug-free remission [20]. The mainstay of medical treatment for JIA typically involves non-steroidal anti-inflammatory drugs (NSAIDs) and/or conventional synthetic disease-modifying antirheumatic drugs (csDMARDs) and/or intra-articular corticosteroid injections (IACIs). Systemic steroids are primarily reserved for systemic JIA (sJIA). The introduction of biologic DMARDs (bDMARDs) began in the early 21st century and have been found to be effective in reducing inflammation and maintaining normal growth [21] and have improved the prognosis of JIA [22]. When comparing patients treated in the methotrexate era to those in the biologic era, a positive impact on disease activity and potential long-term damage was observed with the use of biologics.

According to some research, intra-articular glucocorticoids can hinder the normal growth of the TMJ, and its use can be associated with complications as heterotopic bone formation (HBF) [23–25]. The most recent recommendations for medication of TMJ arthritis are published by American College of Rheumatology (ACR) in year 2021 [26]. The advances in systemic medication, e.g. bDMARDs, have also decreased the use of systemic steroids, thus reducing their negative effects on bone health and children’s growth [27].

The aim of this retrospective study was to evaluate the effectiveness of medical treatments used to manage TMJ arthritis in JIA patients and how to evaluate the treatment outcome. Furthermore, this study aimed to determine the prevalence of TMJ arthritis in JIA patients and investigate the potential impact of specific factors, such as JIA subtype, gender, disease onset, and immunological factors, on this prevalence.

## Methods

### Study population

For this study, we conducted a retrospective analysis of clinical records and imaging studies of JIA patients who had been in treatment between 2015 and 2019 at the Department of Oral and Maxillofacial Diseases, Helsinki University Hospital (HUH), in Helsinki, Finland. The hospital operates in a healthcare district of around 1.8 million residents, with JIA patients in the district being managed and treated by pediatric rheumatologists. To be included in the study, patients had to meet the JIA diagnosis criteria outlined by the ILAR [5]. Patients with incomplete clinical records were excluded (*n* = 22). Overall, 194 patients were included in the study. The length of the patient treatment period at the Department of Oral and Maxillofacial Diseases varied between patients from one visit to about 10 years, depending on the severity of the disease in the TMJ, between the years 2002 and 2019. Data were collected on various factors, such as gender, age, general health, JIA subtype, age at onset of JIA, laboratory results, and medication. Additionally, imaging studies, including panoramic tomographs (PTGs) and magnetic resonance images (MRIs), were evaluated. We used this same study group in our previous study to report JIA patients’ extraoral and intraoral conditions, occlusion and jaw mobility, and presence of orofacial symptoms [28].

### JIA onset definition

The study group was categorized into two subgroups based on the age at which patients were diagnosed with JIA: individuals diagnosed before the age of seven and those diagnosed at or after the age of seven. Patients diagnosed under 7 years of age were categorized in the group ‘early onset’, and patients diagnosed 7 years of age and older in the group ‘late onset’. The division is based on previous research showing that the onset age of JIA peaks during two age ranges: 1–3 years old and 10–12 years old [29]. In addition, children in Finland start going to school at the age of seven.

### TMJ arthritis diagnosis and treatment practices

During the study period, when the pediatric rheumatologists suspected TMJ arthritis, they sent the patient to the dentists (oral and maxillofacial surgeon, prosthodontist, or orthodontist). TMJ arthritis was usually diagnosed via clinical examination and with MRI during dental visits. If the clinical findings were very clear (decreased MIO, swelling, and/or pain in the TMJ), the diagnosis could be established without an MRI examination, especially in polyarticular cases. During the study period there was no validated protocol for orofacial examination.

**Figure 1:**
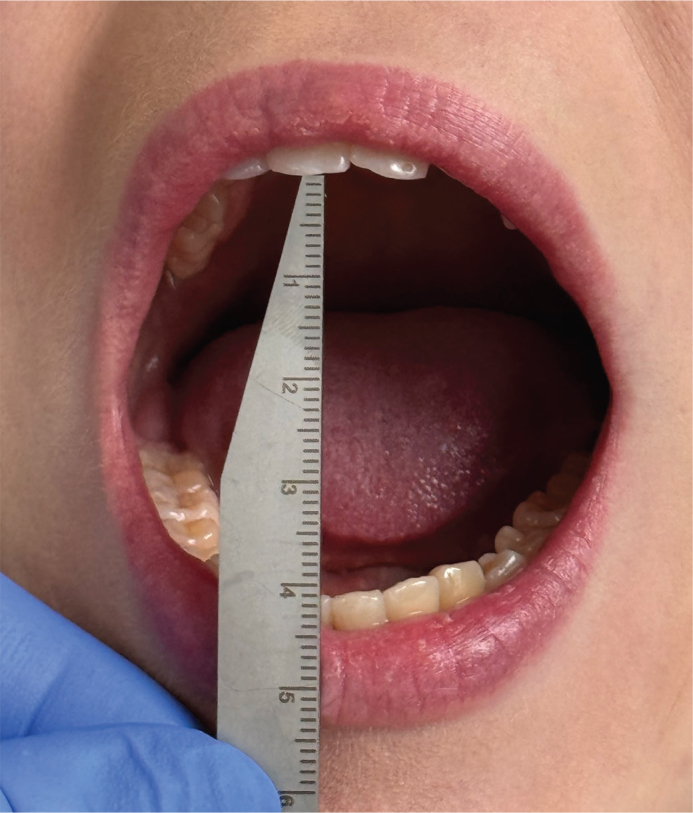
Measurement procedure of MIO. MIO: Maximal incisal mouth opening.

Our practices have developed through clinical work and according to our present protocol, JIA patients under 7 years of age undergo an annual orofacial examination by an orthodontist. Patients with polyarthritis receive annual examinations until they reach the age of 16. Other JIA patients, aged 7 years or more, are sent to orthodontic consultation if needed. Orofacial examination nowadays includes facial examination, occlusal examination, and TMD examination. Facial examination includes examination of profile and asymmetries. Occlusal examination includes angle classification, overbite, overjet, and recording of possible malocclusions such as posterior cross bite, scissors’ bite, crowding, and open bite. TMD-protocol includes measuring MIO, protrusion, lateral movements of the mandible, compression test, deviation of the chin in MIO, and palpation of the jaw muscles. Also, patients were asked about their symptoms in chewing muscles and TMJ. Both pediatric rheumatologists and orthodontists should measure MIO to evaluate the TMJ function. MIO is measured as the vertical distance between the incisal edges of the maxillary and mandibular central incisors (Figure 1). The painless MIO was measured in millimeters (mm).

After TMJ arthritis is diagnosed, IACIs for children under 10–12 years of age are given under general anesthesia by pediatric rheumatologists, and IACIs for children over 10–12 years olds are given under topical anesthesia or nitrous oxide sedation by pediatric rheumatologist or oral and maxillofacial surgeon depending on child’s co-operation. Aftercare instructions advise using the joint minimally on the day in question. After 2–3 months following IACI, an orthodontist checks the joint. If symptoms and clinical findings (decreased MIO, swelling, and/or pain in the TMJ) still exist, a new MRI is taken, and based on the findings of this, systemic medication is augmented and re-IACI is considered. If necessary, in addition to systemic medication and/or IACIs, a dental splint is made and/or physiotherapy prescribed. In this study, MIO was measured 0–3 months before the first IACI and 2–6 months after the first IACI.

### Statistical methods

Categorial variables were reported as percentages. To compare categorial variables, either a Chi-square test or two-proportion *Z*-test was used. Mann–Whitney *U*-test was used to compare the patients’ MIO before and after the first IACI. The statistical analyses were performed using IBM SPSS Statistics for Mac, version 27 (IBM Corp. Armonk, New York, USA) except for *Z*-tests, which were performed using Epitools – Epidemiological calculators (https://epitools.ausvet.com.au/). The level of significance was set at *P* < 0.05.

## Results

### TMJ arthritis and association with JIA subtype, JIA onset age, gender, and immunological findings

The study group consisted of 194 patients: 146 (75%) females, 48 (25%) males. In total, 99 patients (51%) had TMJ arthritis, 71 (72%) female patients, and 28 (28%) male patients. Of all the patients with TMJ arthritis, 70 patients (71%) were diagnosed with MRI in addition to clinical assessment, and 29 patients (29%) were diagnosed clinically by a pediatric rheumatologist using ultrasound as an aid if needed. TMJ arthritis was most common in the oligoarthritis and RF– polyarthritis subgroups (Table 1). The third subgroup (RF+ polyarthritis, psoriatic arthritis, enthesitis-related arthritis, undifferentiated arthritis, systemic arthritis) was named as ‘other’ (Table 2). No statistical difference was found between the subgroups (*P* = 0.422). TMJ arthritis was seen more among males (58%, compared to females 49%) (Table 2). ANA-positive patients exhibited a lower prevalence of TMJ arthritis, HLA-B27 positive exhibited a higher prevalence, not significantly (Table 2). The onset age of JIA did not appear to impact the prevalence of TMJ arthritis, although patients with late onset were observed to have TMJ arthritis slightly more frequently (57%) than those with early onset (47%) (Table 2).

**Table 1:** Different JIA subtypes and TMJ arthritis.

JIA subtypes	Patients with TMJ arthritis *n* = 99		Patients without TMJ arthritis *n* = 95		All *n* = 194	

	*n*	%	*n*	%	*n*	%
Oligoarthritis	53	50	52	50	105	100
Polyarthritis, RF–	41	55	34	45	75	100
Polyarthritis, RF+	0	0	4	100	4	100
Psoriatic arthritis	2	100	0	0	2	100
Enthesitis-related arthritis	2	33	4	67	6	100
Undifferentiated arthritis	1	100	0	0	1	100
Systemic arthritis	0	0	1	100	1	100

JIA: Juvenile idiopathic arthritis; TMJ: Temporomandibular joint; RF: Rheumatoid factor.

**Table 2:** The potential impact of specific factors on TMJ arthritis.

Study variables	Patients with TMJ arthritis *n* = 99		Patients without TMJ arthritis *n* = 95		All *n* = 194		*P*

	*n*	%	*n*	%	*n*	%	
**Gender**							
Female	71	49	75	51	146	100	0.243*
Male	28	58	20	42	48	100	
**Onset age**							
Onset age < 7	52	47	60	53	112	100	0.134*
Onset age ≥ 7	47	57	35	43	82	100	
**JIA Subtype**							
Polyarthritis, RF–	41	55	34	45	75	100	0.422*
Oligoarthritis	53	50	52	50	105	100	
Other	5	36	9	64	14	100	
**Immunological findings**							
ANA+	18	40	27	60	45	100	0.0578^#^
HLA-B27+	22	52	20	48	42	100	0.7139^#^

* Chi-square test used for analysis.

# *Z*-test (Epitools) used for analysis.

JIA: Juvenile idiopathic arthritis; TMJ: Temporomandibular joint; RF: Rheumatoid factor; ANA: antinuclear antibody; HLA-B27: human leukocyte antigen B27.

### Medication

99% of the patients with TMJ arthritis were treated at least once with IACI. In total, 52% of these patients received only one IACI during their treatment period, 35% got two IACIs, 5% got three IACIs, and 8% got four or more IACIs. Patient treatment periods were, in some cases long, so the IACIs were administered over the course of up to 10 years. Also, IACIs given to the left and right TMJ are counted as separate visits if they were not given in the same treatment session.

Before the first IACI, 42 (43%) patients with TMJ arthritis received no systemic medication (Table 3), and after the first IACI systemic medication was started with 26 (16%) of patients. After the second IACI, all patients were receiving systemic medication. Before the first IACI, 18% of patients had received combination drug therapy, while after several IACIs, most of the patients had received combination drug therapy. The number of patients receiving csDMARDs increased with the number of IACIs received: before the fourth IACI, 100% of patients were receiving a csDMARD. Also, the number of patients who had undergone combination drug therapy and/or received a bDMARD increased with the number of IACIs received. The effectiveness of the systemic medication was evaluated each time as IACI was administered, and if necessary, the rheumatologist intensified the medication, for example by increasing the dose, changing the medication, or changing the form of administration.

**Table 3:** Systemic medication during the TMJ IACIs.

Medication	T1 *n* = 98		T2 *n* = 47		T3 *n* = 13		T4 *n* = 8		T5 *n* = 5	

	*n*	%	*n*	%	*n*	%	*n*	%	*n*	%
No systemic medication										
Before	42	43	5	11	1	8	0	0	0	0
After	26	27	0	0	1	8	0	0	0	0
csDMARD										
Before	49	50	33	70	12	92	8	100	5	100
bDMARD										
Before	10	10	7	15	6	46	3	38	4	80
Prednisolone										
Before	17	17	8	17	3	23	2	25	1	20
Combination drug therapy										
Before	18	18	11	23	9	69	5	62	4	80

‘Before’ refers to the time before the IACI and ‘after’ refers to the time after the IACI. IACIs given to the left and right TMJ are counted as separate visits if they were not given in the same treatment session. T_1_: Time of first IACI; T_2_: Time of second IACI, and so on. TMJTMJ: temporomandibular joint; IACI: Intra-articular corticosteroid injections; csDMARD: conventional synthetic disease-modifying antirheumatic drugs; bDMARD: biological drugs.

For comparison, only 7% of all JIA patients in our study received no systemic medication during the research period, and 90% of the patients received a csDMARD at some point of their treatment/disease history, whether they had TMJ arthritis or not.

### TMJ arthritis and MIO after the IACI

**Figure 2:**
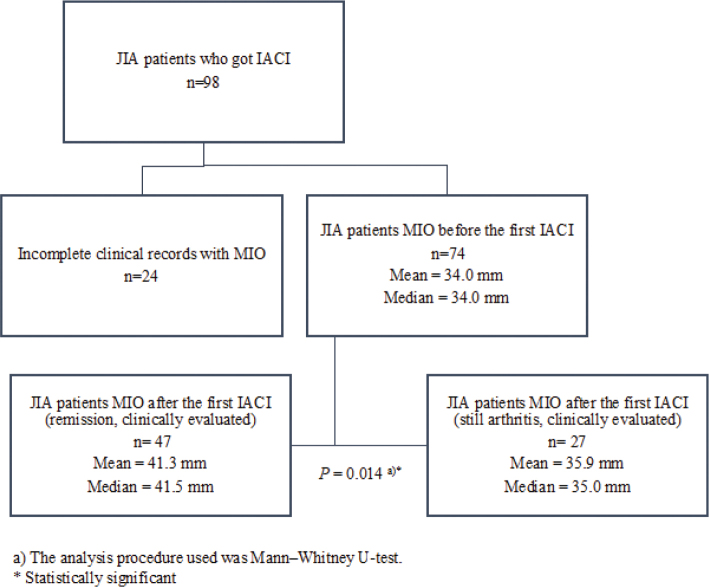
MIO before and after the first IACI. IACI: Intra-articular corticosteroid injections; TMJ: temporomandibular joint; MIO: Maximal incisal mouth opening.

**Figure 3:**
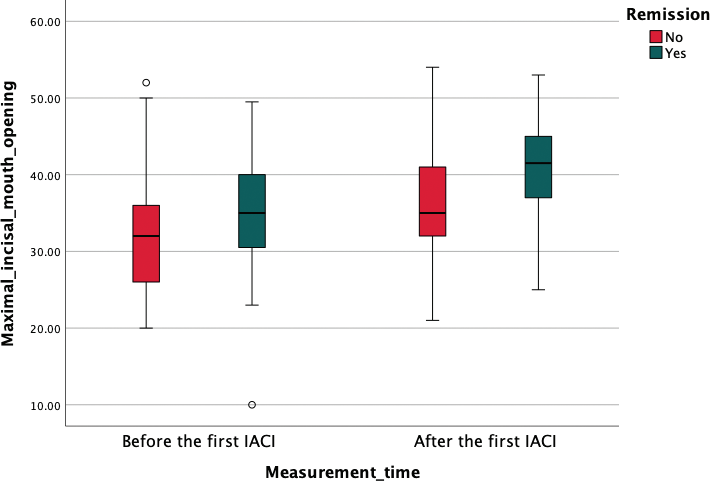
Box plot illustrating MIO (in mm) before and after IACI for the TMJ comparing patient groups who experienced remission in TMJ and those with persisting arthritis in TMJ. MIO was measured 0–3 months before the first IACI and 2–6 months after the first IACI. The central line within each box represents the median value, while the box itself spans the IQR from the 25th percentile to the 75th percentile. The whiskers extend to the minimum and maximum values within 1.5 times the IQR, with outliers depicted as individual points. IACI: Intra-articular corticosteroid injections; TMJ: temporomandibular joint; MIO: Maximal incisal mouth opening; IQR: interquartile range.

If the arthritis persisted in TMJ after the first IACI, patients were treated again with IACI within 6 months from the first IACI or by changing the systemic medication. MIO increased significantly (*P* = 0.014) with patients who achieved remission after the first IACI (median change of 5 interquartile range (IQR) (2; 10)) compared to those whose arthritis persisted after the first IACI (2 (–1; 6)) (Figures 2 and 3). The remission of JIA patients TMJ arthritis was evaluated mostly by clinical methods (based on symptoms, etc.), but in some cases also with MRI.

## Discussion

In our study, half of the JIA patients were diagnosed with TMJ arthritis; nearly two thirds were diagnosed both clinically and by MRI and one third only clinically. Previous studies show great heterogeneity in the way TMJ arthritis is diagnosed. Additionally, the study methods, terminology, and treatment methods vary greatly, and this makes it hard to compare the studies [30, 31]. Only MRI has proven to be reliable enough to diagnose active arthritis in the TMJ [32]. According to current knowledge, active TMJ arthritis cannot be diagnosed based on clinical examination alone [33], because it is difficult to distinguish a clinically active inflammatory process from a chronic condition; this may distort our results. It is also difficult to differentiate discus problems without MRI. A recently published standardization of the terminology for orofacial conditions in JIA suggests using the term TMJ arthritis only if there is proof of an acute inflammation process in the TMJ (in other words, verified by MRI) [31]. This condition is partially fulfilled in our study. Furthermore, similar results on the prevalence of TMJ arthritis have been published by Stoll et al. [12]. In their study, TMJ arthritis was diagnosed by MRI in a study group of 187 JIA patients. TMJ arthritis was detected in 43% of these patients, with no significant differences among different JIA subtypes. This is in line with our results. However, it must be kept in mind that in our study, the study group is selective, and TMJ arthritis was already suspected when the rheumatologist sent the patients to the dentist, so not all the JIA patients were screened.

Early detection of TMJ arthritis is a critical step in preventing damage in the TMJ. To diagnose TMJ arthritis, the clinical orofacial examination should be both extraoral and intraoral. Facial conditions (such as asymmetries) and occlusion should be reported at every visit. Functions such as MIO and deviations, lateral movements, and protrusion are also important. Symptoms such as pain while opening the mouth or a palpating TMJ can indicate TMJ arthritis. Stoll et. al. [12] showed in their study a correlation between decreased MIO and TMJ arthritis. Previous research have also shown MIO improvement after treatment of TMJ arthritis with IACI, and in our study we showed similar results [34]. It is important to keep in mind that MIO is correlated to child’s age [35] and measuring MIO is highly sensitive and requires a standardized measuring protocol [36]. Active arthritis may also have very few symptoms [37], and both decreased mouth opening and greater asymmetries are reported to be associated exclusively to those TMJs in which the deformities already exist [38]. If TMJ arthritis is suspected, PTG may assist the diagnosis, although if changes are seen in PTG, the point of early diagnosis may have already passed [19]. As discussed, the gold standard for diagnosing TMJ arthritis and early detection of inflammatory changes in the TMJ is contrast-enhanced MRI [31, 39].

In our study, male patients had a higher incidence of TMJ arthritis than female patients; however, there was no statistical difference between sexes. This is in contradiction with previous research data. According to a study by Schuckmann et. al. [13], TMJ involvement is more associated with female gender, the oligoarthritis subtype, and ANA positivity. A limitation of this study is that TMJ involvement is not specified, and it is unclear if all the patients had TMJ arthritis. Elsewhere, it has been reported that TMJ arthritis is common among those with the polyarthritis JIA subtype [18]. In our study, we found no statistical significance in the association between JIA subtypes and TMJ arthritis, nor for an association of ANA positivity and TMJ arthritis. Still, despite the statistical non-significance in our study, TMJ arthritis was more common in the oligoarthritis and polyarthritis (RF–) subgroups. Previously, it has been reported that early onset time is also associated with TMJ arthritis [11, 18], but in our study, it was rather late onset patients who had a higher incidence of TMJ arthritis (not statistically significant). These results might be different because of the nature of this retrospective study, but also because of the difference in research methods between the other studies. Furthermore, in our study, some of the subgroups are rather small, and this might affect the results.

In a previous study of ours [28], we reported that late-onset JIA patients report more TMD symptoms compared to early-onset patients, and we proposed that this was due to young children’s inability to verbalize pain. This might still be the case, but the current study points to a real higher incidence of TMJ arthritis in late-onset JIA patients. It may also be that the TMJ symptoms appear when the arthritis has progressed and there already exists destruction in the joint structure.

In our study, almost all patients with TMJ arthritis were treated with IACI at some point. Patients who had many IACIs also had multiple systematic medication before the IACIs, indicating that the use of several systematic medications (history of many different csDMARDs and bDMARDs) alone was not efficient enough to treat TMJ arthritis. Stoll et. al. [12] reported that despite systematic antirheumatic medication, TMJ arthritis might still occur in some patients, and regular control of JIA patient TMJs is needed. Other treatment methods, such as IACIs, might also be needed. TMJ Juvenile Arthritis Working Group (TMJaw) [30] suggest, in a recently published study, that as part of their new treatment protocols and recommendations, the optimal systemic treatment should be considered for active TMJ arthritis in JIA patients. Beneficial effects of systematic immunosuppressive therapy in TMJ arthritis and mandibular growth have been reported [40, 41]. The recommendation by TMJaw also states that IACIs may be used cautiously in patients with refractory TMJ arthritis and orofacial symptoms. Still, caution is needed because it has been reported that the potential hazards associated with inhibiting the growth of the lower jaw and the formation of calcifications within the TMJ may overshadow the positive effects of IACIs for TMJ inflammation, especially in individuals who are still undergoing skeletal development [23, 24]. IACIs are used because they reduce inflammation, so in skeletally mature patients, use of IACIs is recommended if systemic medication alone is not efficient enough to ease TMJ arthritis [30]. Frid et. al. [42] concluded in their study that single IACIs combined with systemic medication is proven to treat inflammation in the TMJ, and no severe side effects were reported in adolescents. Besides TMJaw [30] recommendations, the 2021 ACR also recently published guidelines [26] in which they advise caution against the use of IACIs. Instead, they advise using bDMARDs for patients who have had demonstrated an insufficient response to DMARDs. In our study, we found that even bDMARDs are not always enough. Therefore, more research is needed to map the possible side effects of IACIs and to determine their safe use, especially for skeletally immature patients. To our knowledge, complications had occurred rarely during or after the IACI done in our clinic, but further studies are needed.

### Strengths and limitations

This study’s main strength lies in its sizeable study population and the thorough and organized approach to treating and monitoring all patients with JIA by pediatric rheumatologists at the university hospital. However, as the study was retrospective in nature, some patient data may not have been recorded accurately due to differences in clinician data entry and a lack of standardized examining protocols. Additionally, the long period of patient data collection spanned changes and improvements to the treatment protocol, potentially affecting data accuracy towards the end of the data collection period. For example, nowadays, MRIs are more often taken to confirm TMJ arthritis diagnosis. Because of this, the data is polarized. The data set would have been too small if we had only included patients in the study from the time of the new protocol. Also, some of the JIA subgroups were too small to make any conclusions regarding how the subgroup affects the prevalence of TMJ arthritis.

## Conclusion

- MIO is an easy way to evaluate the treatment outcome and possible disease activation of TMJ arthritis.
- The prevalence of TMJ arthritis is high among JIA patients.
- Parameters that could predict TMJ arthritis were not detected.
- Despite systemic medication, TMJ arthritis might occur, and often there is a need for additional treatments, such as IACIs or systemic medication alterations/intensifications. More research is needed on how safe IACIs are in growing patients.
- It is important that JIA patient TMJs are under regular and systematic monitoring.

## Ethical permission

The Hospital District of Helsinki and Uusimaa approved the protocol for this retrospective study (HUS/037/2019). As the study did not meet the criteria of a medical study as outlined in the Medical Research Act, ethical approval was not required. The principles outlined in the Declaration of Helsinki were followed.

## Data Availability

The data will be shared on reasonable request to the corresponding author.
